# Decreased DNA Repair Ability: A Mechanism for Low Early Embryonic Development Potential of Oocytes From Overweight Patients After Fertilization in IVF Cycles

**DOI:** 10.3389/fendo.2021.756336

**Published:** 2021-11-23

**Authors:** Hui Li, Huan Wang, Jing Zhu, Jianmin Xu, Yuqing Jiang, Wenhui Chen, Yingpu Sun, Qingling Yang

**Affiliations:** ^1^ Center for Reproductive Medicine, The First Affiliated Hospital of Zhengzhou University, Zhengzhou, China; ^2^ Henan Key Laboratory of Reproduction and Genetics, The First Affiliated Hospital of Zhengzhou University, Zhengzhou, China; ^3^ Henan Provincial Obstetrical and Gynecological Diseases (Reproductive Medicine) Clinical Research Center, The First Affiliated Hospital of Zhengzhou University, Zhengzhou, China

**Keywords:** obesity, oocyte, embryo quality, sperm DNA fragmentation (SDF), DNA repair, *in vitro* fertilization

## Abstract

**Background:**

Whether female BMI impacts the DNA repair ability in the oocytes after fertilization has not been investigated. The aim of this study is to assess the early embryo quality and reproductive outcomes of oocytes from overweight women when fertilized with sperm with varying degrees of DNA fragmentation.

**Methods:**

A total number of 1,612 patients undergoing fresh autologous *in vitro* fertilization (IVF) cycles was included. These patients were divided into two groups according to maternal body mass index (BMI): normal weight group (18.5–24.9 kg/m^2^; n=1187; 73.64%) and overweight group (≥25 kg/m^2^; n=425; 26.36%). Each group was then subdivided into two groups by sperm DNA fragmentation index (DFI): low fragmentation group (<20% DFI, LF) and high fragmentation group (≥20% DFI, HF). Laboratory and clinical outcomes were compared between subgroups.

**Results:**

For the normal-weight group, there was no statistical significance in embryo quality and reproductive outcomes between the LF and HF groups. But in the overweight group, significantly lower fertilization rate (LF: 64%; HF: 59%; p=0.011), blastocyst development rate (LF: 57%; HF: 44%; p=0.001), as well as high-quality blastocyst rate (LF: 32%; HF: 22%; p=0.034) were found in the HF group, despite the similar pregnancy rates (LF: 56%; HF: 60%; p=0.630).

**Conclusions:**

Decreased DNA repair activity in oocytes may be a possible mechanism for the low early development potential of embryos from overweight patients in *in vitro* fertilization cycles.

## Introduction

A wide range of disorders were associated with obesity, including chronic low-grade inflammation, metabolic syndrome, diabetes, cardiovascular disease, and infertility (1–4). Recent retrospective population studies have found that obese patients have poor endometrial receptivity (5), poor embryo quality (6, 7), low oocyte maturation rates (8), and subsequently, low pregnancy rate (9, 10) and high abortion rate (11, 12). Nevertheless, the etiology for poor reproductive ability in obese women remains largely unclear. Animal experiments have shown that both gene mutation-induced obesity and high-fat diet (HFD) induced obesity could induce reactive oxygen species (ROS), disrupt meiotic maturation, and affect DNA and histone methylation, as well as decrease DNA repair activity (DRA) in oocytes (13, 14). Moreover, after fertilization, the blastocyst development rate was decreased in HFD-induced obese mice (15). Whether human oocytes exhibit a similar decline in DRA has not been investigated.

Sperm DNA fragmentation index (DFI) is used for evaluating DNA damage and could reflect sperm quality directly (16). During spermatogenesis, fragments of DNA are produced in germ cells when chromosomal or DNA integrity is impaired, including DNA strand breakage, nicks, and deletions (17). Several studies found that no statistical difference was observed in embryo quality and reproductive outcomes between groups with different sperm DFI during IVF cycles (18, 19). However, other studies found that the higher the sperm DFI, the worse the embryo quality and reproductive outcomes are in IVF or intracytoplasmic sperm injection (ICSI) cycles (20–22). Alfonso et al. used a mouse model and demonstrated that both pregnancy rate and live offspring rate were significantly lower when oocytes were fertilized with sperm with high DFI compared with the fresh sperm (23). In addition, they found that ICSI performed with high DFI sperm could lead to effects during aging, such as abnormal growth and premature aging as well as mesenchymal tumors (23). However, the reason for such different results remains unclear. Previous research has shown that oocyte quality and pregnancy outcomes decreased with increasing BMI (24, 25), while the effect of SDF on pregnancy outcome depends not only on the degree of SDF but also on the oocyte quality (26, 27). Given that obesity could induce oocyte dysfunction, we hypothesized that the poor reproductive outcomes of obese women were related to a reduced ability of oocytes to repair DNA damage. Based on these findings and to test this hypothesis, 1,686 patients undergoing fresh autologous IVF cycles in our center were divided into normal weight and overweight groups according to female BMI, then each group was subdivided by sperm DFI into a low fragmentation group (LF) and high fragmentation group (HF). Our results indicate that when fertilized with high DFI sperm, the impaired DNA-repairing ability in the oocyte of overweight patients is at least one cause of poor reproductive outcomes.

## Materials and Methods

### Patient Selection and Study Design

This study collected data from 1,612 fresh autologous IVF cycles from January 2017 to December 2019 in the Reproductive Center of the First Affiliated Hospital of Zhengzhou University. Women were recruited as follows: fresh autologous IVF cycles with females aged ≤40 years and the basal level of follicle-stimulating hormone (FSH) less than 10 mIU/mL. Women with polycystic ovary syndrome (PCOS) were excluded. Couples were excluded when one of them had significant endocrinology or metabolic dysfunctions. Preimplantation genetic tests were also excluded.

Based on the maternal BMI, the patients were divided into two groups: group 1 (18.5≤BMI<25 kg/m2; n=1187; 73.64%), and group 2 containing overweight (25≤BMI<27.9 kg/m2; n=301, 18.67%) and obesity (BMI≥28 kg/m2; n=124, 7.69%). DFI in semen samples was assessed using sperm chromatin structure analysis (SCSA). Each group was then subdivided into two subgroups using the sperm DFI: low fragmentation group (<20% DFI, LF) and high fragmentation group (≥20% DFI, HF). Comparation of embryo quality and pregnancy outcomes between subgroups were analyzed.

### Semen Preparation and Sperm DNA Fragmentation Index

Males remained sexually abstinent for 3–7 days and then collect semen by masturbation. After 30 minutes of liquefaction, the semen sample was used to evaluate standard semen parameters. SDF was measured using SCSA. Specifically, samples were adjusted to a sperm density of 0.5–1.0×10^6^/mL in buffer (0.01 M Tris-HCL, and 0.15 M NaCl, 1 mM EDTA). After staining by acridine orange (AO; pH 6.0) solution, signals of fluorescence were detected by a flow cytometer (BD FACS Canto II) to calculate sperm DFI in at least 5,000 sperm per sample. DNA was denatured by acid treatment and stained with acridine orange (AO). The double-stranded DNA bound with AO emitted green fluorescence, while the single-stranded DNA bound with AO emitted red fluorescence. Further evaluation of the stained cells using flow cytometry showed that the green-stained sperm had intact DNA, while the red-stained sperm had denatured DNA. DFI can be calculated based on the proportion of red sperm count (28).

### ART Treatment

Controlled ovarian stimulation is achieved with ultralong treatment regimens. on the 2nd and 3rd day of menstruation, Injection of gonadotropin-releasing hormone-a (GnRH-a, triptorelin acetate) 3.75 mg to downregulate the gonadotropin (Gn) secretion. After 28 days, blood tests for basic sex hormone levels and ultrasonic examinations were performed. Then, the ovulation induction process will be initiated when the Gn level reached a low threshold. Recombinant-human chorionic gonadotropin (hCG) (Serono, Switzerland) 10,000 IU was used to trigger eventual follicular maturation when two or more follicles reached the diameter of 17 mm. Oocytes were collected for IVF after 35-37 hours.

### Embryo Culture

Embryos were cultured in a sequential culture medium (Vitrolife, Sweden) under 6% CO2 and 37°C. After 16 to 18 hours, fertilization was evaluated by observation of pronuclei. The embryo quality of the cleavage stage was graded using Peter’s scoring system (29) and the blastocyst-stage embryos were graded using the Gardner’s standard (30). Embryo was transferred on Day3 or day5.

### Evaluation of Pregnancy Outcomes

Positive serum β-hCG on 14 days after embryo transfer was considered a biochemical pregnancy. Thirty-five days after transplantation, ultrasound was used to diagnose clinical pregnancy by observing the gestational sac.

### Statistical Analysis

The basic conditions of the patients in the two groups, including paternal age, paternal DFI, maternal age, maternal BMI, basal FSH, LH, P, and E2 levels, and the number of retrieved oocytes and mature oocytes, were presented as mean ± standard deviation. Laboratory indicators and clinical outcomes were expressed by frequency (percentage) and were compared by chi-square analysis. The data was analyzed by SPSS 26.0 software (Version 26.0, IBM Corp., Armonk, NY). A P<0.05 was considered statistically significant.

## Results

A total of 1,612 couples undergoing fresh autologous IVF cycles were enrolled in this study. Patients were divided into two groups based on maternal BMI: group 1 (18.5≤BMI<25 kg/m2; n=1187; 73.64%), and group 2 (BMI≥25 kg/m2; n=425, 26.36%). Basic information for group 1 and group 2 was shown in **Table 1**. There was no statistical difference in maternal age and AMH level between the two BMI groups. Basal hormone level on D3 showed that compared with group 1, the level of FSH (p<0.001) and LH (p=0.006) was significantly lower in group 2, both of them are in the normal range. While there was no statistical difference between group1 and group 2 in the level of basal E2 and P.

**Table 1:** Female clinical basic characteristics in group 1 and group 2.

	18.5 ≤ BMI < 25 n = 1187	BMI ≥ 25 n = 425	P value
Maternal age (y)	31.13 ± 4.22	31.67 ± 4.46	0.088
Maternal BMI (kg/m2)	21.75 ± 1.71	27.22 ± 1.9	0.000
Basal FSH	6.83 ± 1.62	6.38 ± 1.74	0.000
Basal LH	5.52 ± 4.06	4.86 ± 4.6	0.006
Basal E2	45.41 ± 50.36	42.69 ± 48.54	0.326
Basal P	0.52 ± 1.04	0.58 ± 1.31	0.387
AMH	3.22 ± 2.33	2.98 ± 2.07	0.064

Data are presented as mean ± standard deviation (x ± SD). BMI, body mass index; FSH, follicle-stimulating hormone; LH, luteinizing hormone; E2, estradiol. P, progestogen; AMH, anti-Müllerian hormone.

Group 1 and group 2 were then subdivided into two groups by sperm DFI respectively: low fragmentation group (<20% DFI, LF) and high fragmentation group (≥20% DFI, HF). Basic information for the two subgroups is shown in **Table 2**. In group 1, the paternal age was significantly higher in the HF group (LF: 31.74 ± 4.91 years; HF: 32.93 ± 5.22 years; p=0.002) than that of LF group, as was found previously that male SDF increases with age (31). No statistically significant difference was observed in maternal age, BMI, basal FSH, LH, E2, and P levels, oocytes retrieved, and matured oocyte numbers between the two subgroups in each BMI group.

**Table 2:** Baseline characteristics in each subgroup.

	18.5 ≤ BMI < 25 (n = 1187)			BMI ≥ 25 (n = 425)		
	<20% DFI n = 987	≥20% DFI n = 200	P value	<20% DFI n = 368	≥20% DFI n = 57	P value
Paternal age (y)	31.74 ± 4.91	32.93 ± 5.22	0.002	32.28 ± 4.65	33.46 ± 5.37	0.161
Paternal DFI	10.27 ± 4.43	26.86 ± 7.2	0.000	10.48 ± 4.44	28.15 ± 8.25	0.000
Maternal age (y)	31.03 ± 4.12	31.62 ± 4.66	0.096	31.71 ± 4.44	32.37 ± 4.84	0.300
Maternal BMI (kg/m2)	21.73 ± 1.7	21.85 ± 1.75	0.400	27.27 ± 1.91	26.95 ± 1.76	0.218
Basal FSH	6.81 ± 1.61	6.94 ± 1.68	0.301	6.4 ± 1.74	6.26 ± 1.75	0.709
Basal LH	5.58 ± 3.99	5.22 ± 4.37	0.259	4.8 ± 4.7	5.26 ± 3.87	0.168
Basal E2	45.32 ± 48.73	45.86 ± 57.84	0.890	42.06 ± 49.75	46.78 ± 39.99	0.495
Basal P	0.52 ± 1.02	0.55 ± 1.15	0.649	0.6 ± 1.4	0.47 ± 0.38	0.642
Retrieved oocytes (n)	12.8 ± 5.92	12.28 ± 5.72	0.255	12.91 ± 6.77	12.58 ± 6.83	0.665
Mature oocytes (n)	10.25 ± 5.18	9.61 ± 5.2	0.111	10.4 ± 5.95	9.42 ± 5.02	0.373

Data are presented as mean ± standard deviation (x ± SD). BMI, body mass index; DFI, DNA fragmentation index; FSH, follicle-stimulating hormone; LH, luteinizing hormone; E2, estradiol; P, progestogen.

As shown in **Table 3**, no statistical difference was found in embryo quality and reproductive outcomes of the cycles between the HF and LF groups for group 1. When it comes to group 2 (**Table 4**), the fertilization rate (LF: 64%; HF: 59%; p=0.011), lower blastocyst development rate (LF: 57%; HF: 44%; p=0.001), and high-quality blastocysts rates (LF: 32%; HF: 22%; p=0.034) were much lower in HF group than that of LF group. However, no significant difference in the cleavage rate and good quality embryos rate were observed. Similarly, on the premise that there was no statistical difference in the mean number of embryos transferred, the implantation rate, pregnancy rate, live birth rate were also no statistical difference.

**Table 3:** Laboratory and clinical outcomes in group 1 with high and low DFI.

Laboratory and clinical outcomes	<20% DFI (n = 987)	≥20% DFI (n = 200)	P value
Fertilization rate	7891 (0.63)	1486 (0.62)	0.190
Cleavage rate	7781 (0.99)	1465 (0.99)	0.954
Good quality embryos rate	5350 (0.69)	996 (0.68)	0.560
Blastocyst formation rate	2343 (0.56)	418 (0.55)	0.516
Good-quality blastocyst rate	752 (0.32)	119 (0.28)	0.142
Embryos transferred	1.68 ± 0.468	1.69 ± 0.464	0.742
Implantation rate	763 (0.46)	150 (0.45)	0.611
Pregnancy rate	605 (0.61)	126 (0.63)	0.652
Live birth rate	504 (0.51)	106 (0.53)	0.617
Clinical miscarriage rate	86 (0.09)	15 (0.08)	0.575

Data are presented as mean ± standard deviation (x ± SD) or the frequency (percentage).

**Table 4:** Laboratory and clinical outcomes in group 2 with high and low DFI.

Laboratory and clinical outcomes	<20% DFI (n = 368)	≥20% DFI (n = 57)	P value
Fertilization rate	3031 (0.64)	418 (0.59)	0.011
Cleavage rate	2992 (0.99)	413 (0.99)	0.877
Good quality embryos rate	2024 (0.68)	272 (0.66)	0.467
Blastocyst formation rate	961 (0.57)	92 (0.44)	0.001
Good-quality blastocyst rate	312 (0.32)	20 (0.22)	0.034
Embryos transferred	1.61 ± 0.488	1.70 ± 0.464	0.221
Implantation rate	262 (0.44)	44 (0.46)	0.739
Pregnancy rate	207 (0.56)	34 (0.60)	0.630
Live birth rate	208 (0.57)	40 (0.70)	0.052
Clinical miscarriage rate	30 (0.08)	3 (0.05)	0.448

Data are presented as the frequency (percentage).

## Discussion

The current study found that there was no statistical difference in laboratorial and clinical outcomes between HF and LF groups in normal-weight women. In contrast, in overweight women with oocytes of poor quality, significantly lower fertilization rate, blastocyst development rate, and high-quality blastocyst rates were found in the HF group in comparison with the LF group. Based on the findings above, we hypothesized that oocytes from overweight patients have decreased DRA, which leads to lower early embryonic development potential. Our findings are in accordance with a study by Meseguer et al. indicating that when obese women use autologous oocytes for ART, higher sperm DFI results in a lower clinical pregnancy rate, but when donor oocytes with good quality were employed, no statistical difference was observed in the clinical pregnancy rate among different SDF groups (26). Interestingly, many studies about to what extent the sperm DFI affects the clinical pregnancy outcomes in IVF support our hypothesis, although results varied. One previous study showed that in IVF or ICSI cycles, no statistical difference in the fertilization rate, good-quality embryo rate, clinical pregnancy rate, or early abortion rate was observed among different sperm DFI groups (18). Another study observed that in comparison with the low SDF group, the high-quality embryo rate at day 3, blastocyst formation rate, blastocyst quality rate, and implantation rate were significantly decreased in the high SDF group, despite the pregnancy rate was similar between the two groups (20). The major difference in inclusion and exclusion criteria between these two studies was average BMI, with 23 and 25 kg/m^2^ in the former and latter, respectively. Thus, embryo quality and reproductive outcomes may decline as a result of decreased oocyte DRA, which partially depends on obesity.

A previous study performed single-cell RNA-sequencing analysis on murine oocytes and showed that the expression levels of DNA damage repair-related genes in 2-cell embryos from HFD-induced mice were all altered compared with embryos from control mice in IVF (32). In addition, the fluorescence intensity of γH2AX staining was higher in embryos from HFD-induced mice than that of the control group, indicating that the DNA damage of oocytes from obese mice had increased but DNA repair ability was reduced. Yet, it is noteworthy that we currently have no way to directly detect whether DNA damage in an embryo is elevated or not, and how well DNA damage is repaired. Future studies are needed to determine how to examine and potentially increase the DRA in oocytes from overweight women.

The limitation of this study is that the sample size of obese patients was too small. Although the present study showed that for overweight women, increasing sperm DFI caused decreased early embryo development potentials, such as reduced fertilization rate, blastocyst development rate, as well as high-quality blastocyst rate in IVF cycles, no significant difference was found in the clinical pregnancy rate and abortion rate between the high and low sperm DFI groups.

In contrast to the current findings, a prospective study by Renato et al. showed that implantation rate, and clinical pregnancy rate, as well as live birth rate, were decreased in the high sperm DFI group, with no statistical difference in fertilization rate and top-quality embryo rate between high and low sperm DFI groups (33). Other studies have shown that reduced embryo quality was found with a high sperm DFI while the pregnancy rate was similar with either high or low sperm DFI (34). Such results may reflect that some studies only observed the clinical pregnancy rate in a fresh transplant cycle, rather than the cumulative pregnancy rate, leading to a convergence of clinical pregnancy rate results. In addition, there are various methods to detect sperm DFI (28, 35–37). Different detection methods and mechanisms may lead to different results. Also, the grouping thresholds for high and low sperm DFI were different in each study and may lead to different conclusions.

Taken together, the present evidence suggests that a reduced ability to repair DNA damage in oocytes is at least one of the reasons for adverse reproductive outcomes in overweight patients using ART with a high sperm DFI. But the influence of other oocytes factors on reduced IVF outcomes cannot be ruled out. Since sperm with a high DFI can fertilize oocytes (38), it is noteworthy that there is no reliable standard to determine the DNA repair ability in oocytes. In addition, it is hard to confirm to what extent was the sperm DNA damage in the fertilizing one.

## Conclusions

In summary, our results provide a new opportunity to improve embryonic development outcomes in IVF cycles for overweight women. In addition to the acknowledgment that obese women should lose weight for optimal fertility, sperm quality should also be improved. The sperm DFI can be lowered by lifestyle modifications (39), dietary changes (40), and antioxidant supplementation (41) before IVF. Such information should be considered to develop better ART procedure strategies for overweight and obese women.

## Data Availability Statement

The raw data supporting the conclusions of this article will be made available by the authors, without undue reservation.

## Author Contributions

YS and QY contributed to the study design, analysis, and interpretation of data. HL and QY contributed to the manuscript drafting, data analysis, and critical discussion. HW, JZ, JX, YJ, and WC helped to collect data and performed data proofreading. All authors contributed to the article and approved the submitted version.

## Funding

This research was supported by the National Key R&D Program of China 2019YFA0110900 and Key Program for International Science and Technology Cooperation Projects of China 81820108016 to YS and National Natural Science Foundation of China 31970800 to QY.

## Conflict of Interest

The authors declare that the research was conducted in the absence of any commercial or financial relationships that could be construed as a potential conflict of interest.

## Publisher’s Note

All claims expressed in this article are solely those of the authors and do not necessarily represent those of their affiliated organizations, or those of the publisher, the editors and the reviewers. Any product that may be evaluated in this article, or claim that may be made by its manufacturer, is not guaranteed or endorsed by the publisher.

## Abbreviations

LF, low fragmentation group; HF, high fragmentation group; ART, assisted reproductive techniques; IVF, *in vitro* fertilization; BMI, body mass index; DFI, DNA fragmentation index; SDF, sperm DNA fragmentation; HFD, high-fat diet; DRA, DNA repair activity; ICSI, intracytoplasmic sperm injection; AO, acridine orange; FSH, follicle-stimulating hormone; LH, luteinizing hormone; E2, estradiol. P, progestogen; AMH, anti-Müllerian hormone; GnRH-a, gonadotropin-releasing hormone-a; Gn, gonadotropin; hCG, human chorionic gonadotropin.
